# Biological activities of a garlic–*Cirsium setidens* Nakai blend fermented with *Leuconostoc mesenteroides*

**DOI:** 10.1002/fsn3.1032

**Published:** 2019-05-08

**Authors:** Eric Banan‐Mwine Daliri, Sun‐Il Choi, Bong‐Yeon Cho, Hyeon Yeong Jo, Se‐Hun Kim, Ramachandran Chelliah, Momna Rubab, Joong‐Hark Kim, Hyun‐Taek Oh, Ok‐Hwan Lee, Deog‐Hwan Oh

**Affiliations:** ^1^ Department of Food Science and Biotechnology Kangwon National University Chuncheon South Korea; ^2^ Erom R&D Center Erom Co., Ltd Chuncheon‐si, Gangwon‐do South Korea

**Keywords:** anti‐inflammatory, functional beverage, immune stimulation

## Abstract

In this study, we investigated the antioxidant‐ and immune‐stimulating activities of various garlic–*Cirsium setidens* Nakai blends (fermented and unfermented). The levels of S‐allyl cysteine increased by 2.5 times while pectolinarigenin (an anti‐inflammatory compound) increased about six times (from 1.1 ± 0.04 mg/g to 6.70 ± 0.12 mg/g) after the garlic–*Cirsium setidens* Nakai (80:20%, respectively) blend (S4) was fermented with *Leuconostoc mesenteroides* KCTC 13302. The ferric reducing ability and DPPH radical scavenging activities of all the samples increased significantly after fermentation. Ethanolic extracts of the fermented samples significantly enhanced RAW 264.7 macrophage proliferation in a dose‐dependent manner and induced nitric oxide production. Among the samples, S6 and S8 stimulated the highest levels of nitric oxide (NO) production. S6 significantly induced proinflammatory cytokines TNF‐α and IL‐1β as well as an anti‐inflammatory cytokine IL‐10 relative to control. Since the resolution of an infection would require a harmonized interplay of proinflammatory factors and anti‐inflammatory cytokines, consumption of S6 could be helpful in promoting health.

## 1 INTRODUCTION

Over the last decade, the demand for functional foods and beverages has increased in many parts of the world (Ozen, Pons, & Tur, 2012) and the spread of healthy foods throughout the market has blurred the distinction between pharma and nutrition (Eussen et al., 2011). Recent advances in scientific research support the fact that diet may not only fulfill nutritional needs but also exert beneficial effects against diseases such as obesity (Cho et al., 2017), cancer (Lee et al., 2006), and diabetes. Several critical factors including health deterioration due to busy lifestyles, insufficient exercise, increased awareness toward the link between diet and health and a competitive food market have been considered as the key factors propelling the functional food and beverage market (Daliri & Lee, 2015).

The functional activities of healthy foods come from the numerous bioactive compounds they contain such as polyunsaturated fatty acids, bioactive peptides, flavonoids, and phenolic compounds. The raw materials from which functional foods are developed include meat and animal products as well as vegetables. Vegetables such as garlic (*Allium sativum* L.) have been exploited for their health benefits. Garlic is known for its ability to modulate the immune system (Venkatesh, 2018), suppress cancerous cell growth, reduce serum cholesterol levels and reduce the risk of cardiovascular diseases (Madden, Krehbiel, & Clarke, 2017). In addition, garlic has immense antibacterial activity (Ain et al., 2017). *S*‐Allylcysteine (SAC) a bioactive compound produced in large amounts during garlic aging and is responsible for the multiple pharmacological activities of garlic. SAC is formed by the enzymatic hydrolysis of γ‐glutamyl‐*S*‐allyl cysteine by γ‐glutamyl transpeptidase (γ‐GTP) (Kodera et al., 2002).

*Cirsium setidens* Nakai has also been used as a traditional oriental medicine for centuries. It is a perennial herb belonging to the family Compositae and is distributed mainly in the Gangwon Province of Korea (Cho et al., 2017). The plant has been used for treating edema, bleeding, and hemoptysis since it contains bioactive compounds such as hispidulin 7‐O‐neohesperidoside, pectolinarin, luteolin, and apigenin (Thao et al., 2011). Earlier studies have proven the biological effects of *C. setidens* Nakai, including antioxidant properties (Lee, Heo, Li, Lee, & Wang, 2008), hepatoprotective activities (Yoo, Nam, Kim, Choi, & Park, 2008), and activity against nonalcoholic fatty liver disease (Noh et al., 2013).

Fermentation of medicinal foods with lactic acid bacteria has been shown to prevent high fat diet‐induced hepatic steatosis in mice (Lee, Lee, Yu, Lee, & Cho, 2017) and improve anticancer activities (Kim et al., 2018). There is, however, limited information on whether a fermented blend of garlic and *C. setidens* Nakai beverage would result in any health effects. In the quest to prepare a functional beverage from a combination of garlic and *C. setidens* Nakai, we ascertained the antioxidant‐ and immune‐enhancing abilities of various blends of garlic and *C. setidens* Nakai to find which blend had the best antioxidant activity, cell proliferation ability, nitric oxide‐stimulating activity, and the cytokines the best blend induces.

## 2 MATERIALS AND METHODS

### 2.1 Chemicals and reagents

Trichloroacetic acid (TCA), gallic acid, De Man, Rogosa and Sharpe (MRS) broth, Dulbecco's modified Eagle's medium (DMEM), fetal bovine serum (FBS), and Folin–Ciocalteu's reagent were procured from Sigma‐Aldrich, Inc. Dinitrophenyl hydrazine (DNPH) was from ACROS Organics; hydrogen peroxide, methanol, and FeCl_3_ were purchased from BDH Chemicals Ltd.; and thiourea, CuSO_4_.5H_2_O, H_2_SO_4_, sodium carbonate, AlCl_3_, potassium acetate, Tris–HCl buffer, FeSO_4_, potassium ferricyanide, and ferric chloride were of analytical grade while the water was glass‐distilled. Lipopolysaccharide (LPS), sodium nitrite (NaNO_2_), and Griess reagent (0.1% N‐(1‐naphthyl)ethylenediamine dihydrochloride, 1% sulfanilamide in 5% phosphoric acid) were all prepared in‐house from reagents purchased from Sigma‐Aldrich Korea. Viscozyme, cellulase, amylase, and protease were supplied by Erom Company Limited.

### 2.2 Cell culture

#### 2.2.1 Bacteria culture

*Leuconostoc mesenteroides* KCTC 13302 was obtained from the Department of Food Science and Biotechnology, Kangwon National University, Chuncheon, Korea, and grown in MRS broth for 36 hr at 37°C. The culture was centrifuged at 6,000 g for 15 min to obtain the cell pellets and washed twice with double‐distilled water. The cells were then diluted with distilled water and stored at 4 degrees until use.

#### 2.2.2 Animal cell culture

RAW 264.7 cells (a mouse macrophages cell line) were purchased from the American Type Culture Collection and cultured in DMEM supplemented with FBS (10%), D‐glucose (3.5 mg/ml), sodium pyruvate (100 mM), L‐glutamine (2 mM), penicillin (100 U/ml), streptomycin (100 μg/ml), and amphotericin B (250 μg/ml) at 37°C and 5% CO_2_.

### 2.3 Sample preparation

Garlic (*Allium sativum*) and *C. setidens* Nakai were obtained from Erom Company Limited, Korea. *Leuconostoc mesenteroides* KCTC 13302 was chosen for the fermentation process because it produced the strongest biologically active garlic product in our previous experiments (data not shown).

### 2.4 Preparation of garlic and *Cirsium setidens* Nakai

Garlic was peeled, washed, and dried in a laminar flow hood (Thermo Fisher Scientific) and grounded using a Vitamix 5200 blender (Vita‐Mix Corporation). A portion (100 g) of the ground garlic was weighed with an Ohaus Scout electronic balance (Flinn Scientific) and mixed with 250 ml of distilled water. The mixture was steamed at 100°C for 1 hr and allowed to cool to 50°C. Cellulase (0.5 ml) was added amidst stirring for 30 min after which 0.75 ml of protease was added for 1 hr. Amylase (1.25 ml) was then added and heated for 1 hr at 70°C. The enzymes were then inactivated by increasing the temperature to 90°C for 30 min, and the mixture was concentrated to obtain 7°Bx. The sample was concentrated to 17% (v/v). The sample was adjusted to 10°Bx and freeze‐dried.

For *C. setidens* Nakai preparation, the plant material was extracted with water twice at 90°C and filtered. The sample was then concentrated to 10°Bx, freeze‐dried, and stored at −20°C until use. Different proportions of garlic and *C. setidens* Nakai were mixed in the ratios 90:10, 80:20, 50:50, and 100:0, respectively, and autoclaved.

### 2.5 Preparation of fermented garlic–*Cirsium setidens* Nakai blend

One portion of the different ratios of garlic and *C. setidens* Nakai (90:10, 80:20, 50:50) were inoculated with 0.1% *L. mesenteroides* KCTC 13302 (v/v) in distilled water. The sample was incubated at 37°C for 48 hr (Table 1). The sample was then sterilized by heating at 100°C for 1 hr and concentrated to 30°Bx. The final pH was between 5.4 and 5.7. All the samples (both fermented and unfermented) were freeze‐dried and extracted with 99.9% (v/v) ethanol. The extracts were stored at −20 degrees till use.

**Table 1:** List of samples and their description

Sample number	Description
S1	*Cirsium setidens* Nakai
S2	Unfermented Garlic
S3	*C. setidens* Nakai (10%) + Garlic (90%)
S4	*C. setidens* Nakai (20%) + Garlic (80%)
S5	Fermented S3
S6	Fermented S4
S7	*C. setidens* Nakai (50%) + Garlic (50%)
S8	Fermented S9

### 2.6 Determination of bioactive compounds

#### 2.6.1 S‐allyl cysteine levels by high‐performance liquid chromatography (HPLC)

The levels of S‐allyl‐L‐cysteine were analyzed by the Korean Food Research Institute (Chonbuk). Briefly, the extracts were filtered through a 0.45‐m syringe filter (Merck KGaA) and the filtrate was analyzed using S‐allyl‐L‐cysteine (≥98%; Sigma‐Aldrich) as a standard. A HPLC‐UVD system (Shimadzu, Shimadzu Corporation) fixed with a LC‐10AD pump, a SPD‐10A UV/Vis detector, a CTO‐10AC column thermostat, and a manual sample injector was used to analyze S‐allyl cysteine content in extracts. The mobile phase consisted of 0.1% H3PO4 solution and acetonitrile (Sigma‐Aldrich) with isocratic elution. A flow rate of 0.5 ml/min and injection volume of 10 µl were applied. The analyte was separated using a LiChroCART® column (250 × 4 mm, 5 m, Merck KGaA) at room temperature, and SAC was detected at 210 nm.

#### 2.6.2 Determination of pectolinarin and pectolinarigenin

##### Extraction and isolation

Dried samples (200 g) of *C. setidens* were extracted with water, filtered, and freeze‐dried to obtain 20 g of the dry extract. All the samples (single and blends) were dissolved in methanol and filtered using a 0.45‐μm syringe filter and analyzed using HPLC. A Waters Spherisorb^®^ INNO column C18 (4.6 × 250 mm, 5 μm) was used for analysis of pectolinarin and pectolinarigenin. The mobile phase was dissolved in water (solvent A) and acetonitrile (solvent B). The gradient solvent system was initially composed of solvents A/B (75:25) and then changed to solvents A/B (10:90) for 30 min, solvents A/ B (0:100) for 20 min, and finally to solvents A/B (75:25) for 15 min. The injection volume was 10 μL, and flow rate was 1 ml/ min. The UV spectra were recorded at 254 nm for quantification of flavonoids. All injections were performed in triplicate.

##### Limit of detection (LOD) and limit of quantification (LOQ)

Validation of the HPLC method for pectolinarin and pectolinarigenin as standard compounds was determined by LOD and LOQ. Method linearity was established by triplicate injections in the range of 0.0001−1.0 mg/ml. Five calibration solutions were injected in triplicate. Calibration curves were constructed by linear regression of the peak height (Y) of pectolinarin and pectolinarigenin versus concentration (X) in mg/ml. The relative standard deviation was used as a measure of repeatability. The percent recoveries were evaluated by calculating the ratio of amount detected versus amount added. LOD and LOQ values were determined separately at signal‐to‐noise ratios (S/N) of 3 and 10, respectively.

##### Calibration curves

Stock solutions (1 mg/ml) of pectolinarin and pectolinarigenin were prepared in MeOH, and the solution content was successively reduced to 10% in order to create different concentrations for calibration curves. The calibration curves for pectolinarin and pectolinarigenin were calculated using peak area (Y), concentration (X, mg/ml), and mean value (*n* = 3) ± *SD* (Table 2).

**Table 2:** Total pectolinarin and pectolinarigenin content in samples

Sample	Pectolinarin (mg/g)	Pectolinarigenin (mg/g)
S1	ND	3.70 ± 0.07^a^
S2	ND	ND
S3	ND	0.4 ± 0.14^d^
S4	ND	1.1 ± 0.04^e^
S5	3.68 ± 0.04^a^	2.50 ± 0.04^f^
S6	7.50 ± 0.09^b^	6.70 ± 0.12^g^
S7	ND	1.73 ± 0.14^hr^
S8	10.56 ± 0.02^c^	8.49 ± 0.17^i^

Note

Values represent means of three replicates ± *SD* Bars with different letters are significantly different (*p* < 0.05).

Abbreviation: ND, not detected.

### 2.7 Antioxidant activities

#### 2.7.1 Determination of total phenol content

The total phenolic content was determined according to the method of Singleton, Orthofer, and Lamuela‐Raventós (1999) with slight modifications. Samples of the extracts (200 mg) were dissolved in 1 ml of distilled water and filtered, and 100 μl was oxidized with 2.5 ml of 10% Folin–Ciocalteu's reagent (v/v) inside a test tube. The samples were then neutralized by adding 2.0 ml of 7.5% sodium carbonate. The reaction mixture was incubated for 40 min at 45°C, and the absorbance was measured at 765 nm with an Eppendorf Biospectrometer (Eppendorf Biospectrometer® fluorescence, Eppendorf Korea Ltd.). The total phenolic content of the beverages was subsequently estimated from a standard curve of absorbance of gallic acid and reported as gallic acid equivalent (GAE) (Table 3).

**Table 3:** Total phenolic and total flavonoid contents

Sample	Total phenolic contents (mg GAE^a^/g)	Total flavonoid contents (mg QE^b^/g)
S1	35.10 ± 0.53^a^	28.11 ± 0.18^d^
S2	21.30 ± 0.17^h^	26.1 ± 0.10^e^
S3	21.59 ± 0.14^h^	25.16 ± 0.27^f^
S4	20.11 ± 0.19^i^	26.22 ± 0.57^e^
S5	18.91 ± 0.17^j^	28.29 ± 0.27^d^
S6	23.14 ± 0.19^g^	31.90 ± 0.54^c^
S7	25.10 ± 0.07^f^	27.71 ± 0.04^d^
S8	27.81 ± 0.34^d^	33.80 ± 0.44^b^

Note

Values represent means of three replicates ± *SD*. Values in columns with same alphabets are not significantly different (*p* > 0.05).

a Gallic acid equivalent.

b Quercetin equivalent.

#### 2.7.2 Total flavonoid content

The total flavonoid content of the extracts was determined by the aluminum chloride colorimetric method (Chang, Yang, Wen, & Chern, 2002). In brief, 50 μl of the samples (1 mg/ml ethanol) was made up to 1 ml with methanol, mixed with 4 ml of distilled water and then 0.3 ml of 5% NaNO_2_ solution; 0.3 ml of 10% AlCl_3_ solution was added after 5 min of incubation, and the mixture was allowed to stand for 6 min. Then, 2 ml of 1 M NaOH solution was added, and the final volume of the mixture was brought to 10 ml with double‐distilled water. The mixture was allowed to stand for 15 min, and absorbance was measured at 510 nm. The total flavonoid content was estimated from a calibration curve, and the result was expressed as milligram quercetin equivalent (QE) per g dry weight (Table 3).

#### 2.7.3 Determination of reducing property (FRAP)

**Figure 1:**
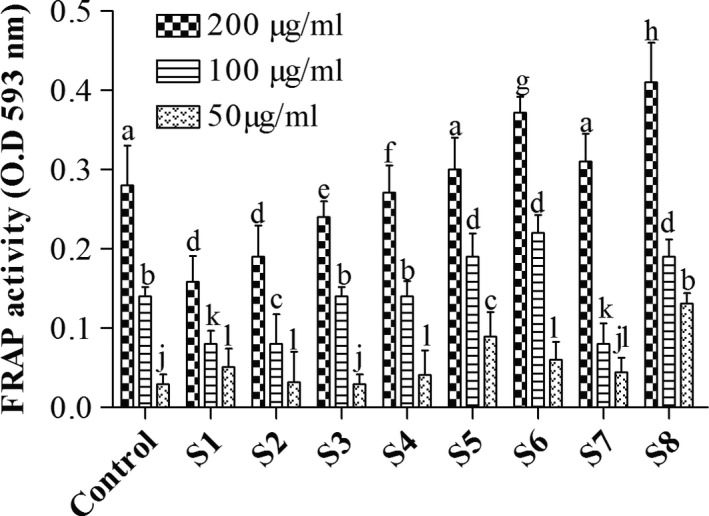
Dose‐dependent FRAP activity of various extracts. Data show mean + *SD* (*n* = 3). Values with different alphabets across a treatment are significantly different (*p* < 0.05) according to Duncan multiple range test

The reducing properties of the extracts were determined by assessing the ability of the extracts to reduce FeCl3 solution as described by Zhao et al. (2008). An aliquot (2.5 ml) of the aqueous extract was mixed with 2.5 ml 200 mM sodium phosphate buffer (pH 6.6) and 2.5 ml 1% potassium ferricyanide. The mixture was incubated at 50°C for 20 min, and then, 2.5 ml 10% trichloroacetic acid (v/v) was added. This mixture was centrifuged at 45 g for 10 min, and 5 ml of the supernatant was mixed with an equal volume of water followed by the addition of 1 ml 0.1% ferric chloride (w/v). The absorbance was measured at 593 nm with an Eppendorf Biospectrometer. Ascorbic acid was used as a positive control. The ferric reducing antioxidant property was subsequently calculated (Figure 1).

#### 2.7.4 2,2‐diphenyl‐1‐picrylhydrazyl free radical scavenging ability (DPPH)

**Figure 2:**
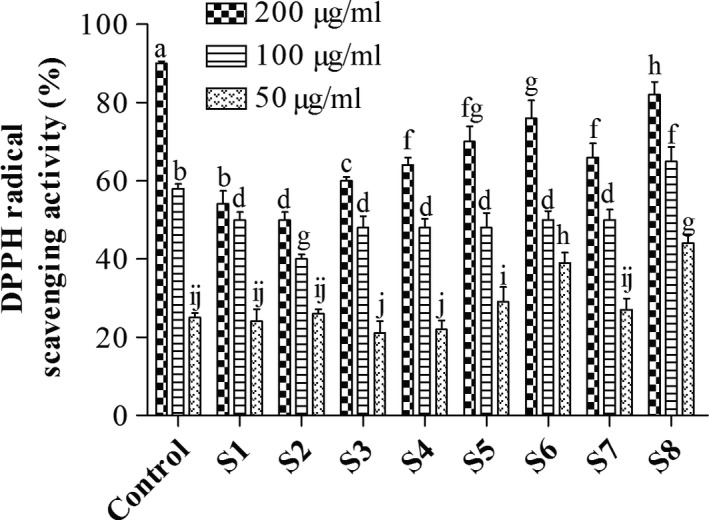
Dose‐dependent DPPH radical scavenging activity of various extracts. Data show mean + *SD* (*n* = 3). Values with different alphabets across a treatment are significantly different (*p* < 0.05) according to Duncan multiple range test

Hydrogen atom or electron‐donation ability of the beverage extracts was measured from the bleaching of the purple‐colored methanol solution of DPPH. The free radical scavenging abilities of the extracts against DPPH (1,1‐diphenyl‐2‐picrylhydrazyl) free radical were evaluated as already described (Gyamfi, Yonamine, & Aniya, 1999). The extracts (200 mg) were dissolved in 1 ml of 0.4 mM methanolic solution containing DPPH radicals. The mixtures were left in the dark for 30 min, and absorbance was taken at 516 nm with an Eppendorf Biospectrometer. Radical scavenging ability of samples was calculated as the percentage of DPPH free radicals inhibited by samples compared to radical inhibition in the negative control (water). Ascorbic acid (AsA) was used as a positive control. The percent DPPH inhibition was calculated from the following equation:

$$\text{DPPH scavenging effect}\;\left(\%\text{inhibition}\right)=\left(\left(A_{\text{control}}-A_{\text{sample}}\right)/A_{\text{control}}\right)\times 100$$

where *A*_0_ is the absorbance of the control and *A*_sample_ is the absorbance of the test samples (Figure 2).

### 2.8 Immunomodulatory activity

#### 2.8.1 RAW 264.7 cell proliferation effects of extracts

**Figure 3:**
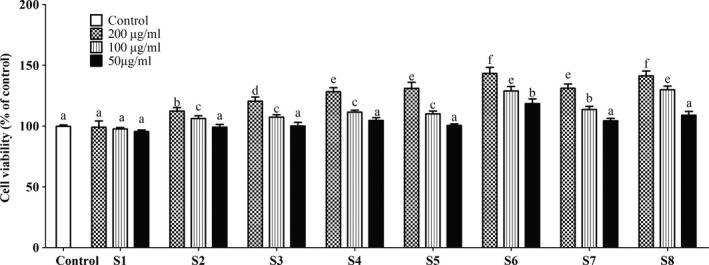
Effects of beverage blends on RAW 264.7 cell proliferation. RAW 264.7 macrophages were either treated with 50, 100, and 200 µg/ml of extracts or were given no treatments (control). Results are expressed as means ± *SD* of three experiments for separate experiments for each data point. Bars with different alphabets are significantly different (*p* < 0.05)

The ability of the extracts to enhance or suppress RAW 264.7 cell proliferation was evaluated by XTT{2,3‐bis (2‐methoxy‐4‐nitro‐5‐sulfophenyl)‐2H‐tetrazolium‐5‐carboxanilide innersalt} assay kit (Welgene Inc). RAW 264.7 cells were seeded in 96‐well plates at a concentration of 1 × 10^6^ cell in 89% Dulbecco's modified Eagle's medium (DMEM from Thermo Fisher Scientific Solutions LLC) containing 10% fetal bovine serum (v/v) and cultured until 100% confluence. Each sample was diluted to 50, 100, and 200 μg/ml. XTT reagent (1 ml) and 20 μl PMS reagent (N‐ethylphenazonium methyl sulfate) were used to prepare a working solution. Equal volumes of the cultured cell supernatant and the working solution (100 μl) were put into the 96‐well plate and were incubated for 4 hr in a CO_2_ incubator (Thermo Scientific). Absorbance was measured using a microplate reader (Tecan GENios FL Fluorescence Microplate Reader FI TRF, USA). The absorbance at 450 nm was subtracted from the absorbance at 690 nm. The ability of the extracts to promote or suppress the growth of the cells was interpreted as their cytotoxicity (Figure 3).

#### 2.8.2 Nitric oxide stimulation assay

**Figure 4:**
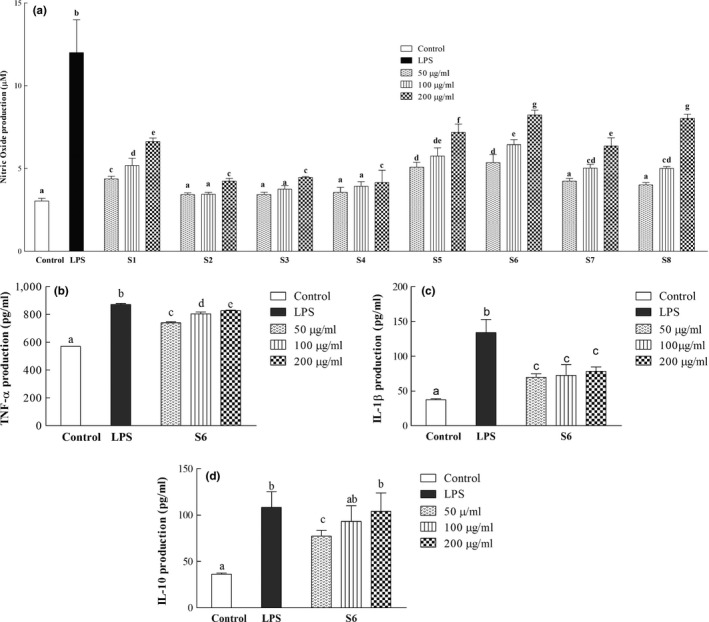
(a) Nitric oxide (NO) production in supernatants of RAW 264.7 macrophages cell culture after beverage extract treatment. The bars represent means of three independent experiments ± *SD*. Bars with the same alphabets are not significantly different (*p* > 0.05). (b) TNF‐α production in supernatants of RAW 264.7 macrophages cell culture after S6 extract treatment. The bars represent means of three independent experiments ± *SD*. Bars with the same alphabets are not significantly different (*p* > 0.05). (c) IL‐1β production in supernatants of RAW 264.7 macrophages cell culture after S6 extract treatment. The bars represent means of three independent experiments ± S.D. Bars with the same alphabets are not significantly different (*p* > 0.05). (d) IL‐10 production in supernatants of RAW 264.7 macrophages cell culture after S6 extract treatment. The bars represent means of three independent experiments ± *SD*. Bars with the same alphabets are not significantly different (*p* > 0.05)

The ability of the extracts to stimulate NO production in RAW 264.7 cells was performed as previously described (Green et al., 1982). Briefly, RAW 264.7 cells (99 μl, plated at 10^6^ cells/ml) were treated with extracts (1 μl). Nitrite was then measured after 24 hr using the Griess reaction. The culture media of the RAW 264.7 cells (80 μl) were mixed with 80 μL of Griess reagent, and its absorbance was measured at 550 nm using an Eppendorf Biospectrometer. The nitrite concentrations in the culture media were determined by comparing them with a NaNO_2_ standard curve. LPS (1 μl) was diluted with DMEM, (0.05 μg/ml) and used as the positive control, and distilled water was the solvent control. Each concentration was assayed three times (Figure 4a).

#### 2.8.3 Cytokine measurements in RAW 264.7 cell cultures

The ability of S6 extract to induce TNF‐α, IL‐1β, and IL‐10 production in RAW 264.7 cells cultures was performed as previously described by with modifications (D'Apolito, Campanozzi, Giardino, & Pettoello‐Mantovani, 2017). Briefly, RAW 264.7 cells (99 μl, plated at 10^6^ cells/ml) were treated with 50, 100, and 200 µg/ml of S6 extracts, and the plates were incubated for 5 days at 37 ºC. LPS (1 μL) was diluted with DMEM, (0.05 μg/ml) and used as the positive control, and distilled water was the solvent control. Each concentration was assayed three times (Figure 4). After incubation, the culture supernatants were harvested, and TNF‐α, IL‐10, and IL‐1β levels were determined using commercial enzyme‐linked immunosorbent assay kits (R&D Systems, Minneapolis, MN) according to the manufacturer's instructions. The results are expressed in pg/ml as mean ± standard deviation.

### 2.9 Statistical analysis

All experiments were carried out in triplicates, and the results were expressed as the mean  ± *SD*. The statistical analysis of data was performed using GraphPad Prism 5.0 (2007) statistical software system (GraphPad Software Inc.). *p* ≤ 0.05 was considered significant according to Duncan multiple range test.

## 3 RESULTS AND DISCUSSION

### 3.1 S‐allyl cysteine, pectolinarin, pectolinarigenin, phenolic, and flavonoid contents

Phenolic compounds are abundant plant secondary metabolites and are beneficial for human health. The phenolic contents of plants have been shown to be directly related to their potential antioxidant capacities in many studies (Demir, Yildiz, Alpaslan, & Hayaloglu, 2014; Zhang et al., 2014). Garlic is rich in phenolic compounds and SAC which have strong antioxidant properties (Alarcón‐Flores, Romero‐González, Vidal, & Frenich, 2014) as well as antihypertensive, anti‐inflammatory, and anticancer activities (Fratianni et al., 2016). *C. setidens* Nakai contains pectolinarin (a glycoside) and pectolinarigenin (an aglycone) which have strong anti‐inflammatory properties (Jeong et al., 2013; Lee et al., 2014). These anti‐inflammatory compounds are, however, bound and are not bioavailable as shown in Table 2. Therefore, fermenting them with *L. mesenteroides* enabled the release of pectolinarin and pectolinarigenin. This accounts for the high pectolinarin and pectolinarigenin contents in fermented samples relative to the unfermented samples containing *C. setidens* Nakai. Fermentation of garlic also significantly increased the levels of SAC from 1.8 ± 0.1 mg/g to 4.43 ± 0.67 mg/g (Table 4). Furthermore, phenolic and flavonoid compounds generally increased with fermentation (Table 3). Our results agree with an earlier study in which the phenolic content of fermented okra seeds increased significantly relative to unfermented seeds (Adetuyi & Ibrahim, 2014). This might be due to the ability of the LAB enzymes to hydrolyze the samples to release bound phenolic and flavonoid compounds during the fermentation process.

**Table 4:** Levels of S‐allyl cysteine in raw and fermented garlic

Sample	S‐allyl cysteine (mg/g)
Raw garlic	1.8 ± 0.1
Fermented garlic	4.43 ± 0.67

### 3.2 Antioxidant activity

Since phenolic and flavonoid contents of plants have been associated with their antioxidant potencies, the Fe^3+^ reducing abilities of the samples were tested (Figure 1). The reducing ability of a compound generally depends on the presence of reductones which exert the antioxidant activity by breaking the free radical chain via donating a hydrogen atom (Pavithra & Vadivukkarasi, 2015). Results from this study demonstrated that sample S8 had a higher Fe^3+ ^to Fe^2+ ^reducing ability followed by S6, and this could be due to the reductones released during the fermentation process.

DPPH, a stable free radical, accepts hydrogen radicals or electrons from donors to become stable diamagnetic molecules. The degree to which a sample decolorizes the dark color of the DPPH radical solution indicates its scavenging potential. In this study, all the extracts scavenged DPPH radicals in a dose‐dependent manner (Figure 2). Consistent with our observations in the Fe^3+^ reducing ability test, sample S8 showed a very high DPPH scavenging activity followed by S6. Since the total polyphenols and flavonoids in the fermented samples increased relative to their unfermented counterparts (Table 3), the high DPPH radical scavenging activity and reducing ability observed could be attributed to the polyphenols and flavonoids released during the fermentation process.

### 3.3 Effects of sample extracts on RAW 264.7 cell proliferation

To test for the potential of the extracts to enhance or suppress cell proliferation, XTT{2,3‐bis (2‐methoxy‐4‐nitro‐5‐sulfophenyl)‐2H‐tetrazolium‐5‐carboxanilide innersalt} assay was performed on RAW 264.7 cells (Figure 3). It was observed that none of the extracts at the concentrations tested suppressed RAW 264.7 cell proliferation. Generally, all the extracts enhanced cell proliferation in a dose‐dependent manner implying that the extracts are nontoxic. The highest cell proliferation was, however, observed after RAW 264.7 cells were treated with 200 µg/ml of S6 and S8. Treating the cells with 200 µg/ml of S6 and S8 resulted in no significant differences in cell proliferation (*p* > 0.05). Lu et al. (Lu et al., 2014) have reported that 50 µM of pectolinarigenin effectively suppresses the growth of MCF‐7 cells (a breast cancer cell line), and our study has shown that the extracts are not toxic to normal cells. This therefore implies that our beverage is safe for consumption and may inhibit tumor growth.

### 3.4 Immunomodulatory activity

To test the ability of the beverage blends to stimulate the immune system, their ability to induce NO production in RAW 264.7 macrophages was studied as these cells constitute the first line of host defenses against infections (Mehta, Ashkar, & Mossman, 2012). When NO is generated, it is easily oxidized to nitrite and stored in intracellular and extracellular fluids (Green et al., 1982). Thus, in experiments, the levels of nitrite (a stable product of NO) are usually measured to reflect the amount of NO (Green et al., 1982). In macrophages, macrophage‐inducible NO synthase is mainly responsible for NO production in response to various stimuli (Tripathi, Tripathi, Kashyap, & Singh, 2007). NO then acts as an intercellular messenger and is a versatile player in the immune system. In our study, all the concentrations tested induce NO to various extents in a dose‐dependent manner (Figure 4a). S8 and S6 induced similar levels (the highest levels) of NO in the culture supernatant. Even lower concentrations (100 μg/ml) of S6 and S8 induced NO production and this agrees with an earlier study which reported that 100 μg/ml of a plant extract strongly induced macNOS mRNA expression (Imanishi et al., 2004). The ability of the extracts to induce NO secretion by RAW 264.7 cells may reflect the potential to modulate NO‐based strategies for pathogen‐mediated immune response for treating infections and tumors.

Due to the high cost of *C. setidens* Nakai, it was more economical to produce S6 which contained only 20% *Cirsium setidens* Nakai relative to S8 which contained 50% *C. setidens* Nakai. Therefore, since they showed comparable effects (cell proliferation ability and NO stimulation), only S6 was selected for further studies.

Activated macrophages are known to produce proinflammatory cytokines such as TNF‐alpha and IL‐1β which play critical roles in regulating immune cells. Both cytokines induce fever, inflammation, and apoptosis and inhibit tumorigenesis and viral replication (Arango Duque & Descoteaux, 2014). For this reason, we measured the levels of TNF‐alpha produced after RAW 264.7 cells were treated with S6 (Figure 4b). TNF‐alpha levels were significantly increased in a dose‐dependent manner relative to the control (*p* < 0.05). However, although IL‐1β levels increased (relative to the control treatment), they were not significantly different (*p* > 0.05) when the concentrations of S6 were increased (Figure 4c).

Activated macrophages can also produce IL‐10 which plays a key role in limiting immune‐mediated pathology during many diseases. This anti‐inflammatory cytokine ameliorates the excessive production of TNF‐α which can result in immunopathology during infections (Couper, Blount, & Riley, 2008). In this study, treatment of RAW 264.7 cell cultures with S6 extracts induced the production of IL‐10 in a dose‐dependent manner (Figure 4d).

The current study has demonstrated the strong antioxidant‐ and immune‐stimulating abilities of a healthy beverage developed by fermenting a blend of *C. Setidens* Nakai and garlic. Since the resolution of an infection would require a coordinated response in which initial proinflammatory factors (such as TNF‐α and IL‐1β) clear the pathogen and (the proinflammatory factors) are subsequently down‐modulated by anti‐inflammatory cytokines such as IL‐10, consumption of S6 could be a good functional beverage for promoting health.

## CONFLICT OF INTEREST

The authors declare that they have no conflict of interest.

## ETHICAL STATEMENT

The study did not involve any human or animal testing.
